# Polycystic Ovarian Condition May Be a Risk Factor for Ovarian Tumor Development in the Laying Hen Model of Spontaneous Ovarian Cancer

**DOI:** 10.1155/2018/2590910

**Published:** 2018-11-25

**Authors:** Hyun Ju Lee, Janice M. Bahr, Pincas Bitterman, Sanjib Basu, Sameer Sharma, Jacques S. Abramowicz, Animesh Barua

**Affiliations:** ^1^College of Pharmacy, University of Illinois at Chicago, IL, USA; ^2^Department of Animal Sciences, University of Illinois at Urbana-Champaign, IL, USA; ^3^Department of Pathology, Rush University Medical Center, Chicago, IL, USA; ^4^Department of Preventive Medicine, Rush University Medical Center, Chicago, IL, USA; ^5^Department of Obstetrics and Gynecology, Rush University Medical Center, Chicago, IL, USA; ^6^Department of Obstetrics and Gynecology, University of Chicago, IL, USA; ^7^Deaprtment of Cell & Molecular Medicine, Rush University Medical Center, Chicago, IL, USA

## Abstract

Chronic inflammation and long-standing oxidative stress are potential predisposing factors for developing malignancies, including ovarian cancer (OVCA). Information on the association of ovarian chronic abnormal conditions, including polycystic ovarian syndrome (PCOS), with the development of OVCA is unknown. The goal of this study was to examine if polycystic ovarian conditions are associated with OVCA development. In the exploratory study, 3–4-year-old laying hens were randomly selected and examined for the presence of polycystic ovaries with cancer (PCOC). In the prospective study, hens were monitored by ultrasound scanning to detect the incidence of a polycystic ovaries and subsequent development of OVCA. Tissues from normal ovaries and PCOC were examined for macrophage infiltration, expression of interleukin-16, and superoxide dismutase 2. The exploratory study detected spontaneous PCOC at early and late stages in hens. PCOC in hens were accompanied with influx of macrophages (17.33 ± 2.26 in PCOC at the early stage and 24.24 ± 2.5 in PCOC at the late stage in 20 mm^2^ areas of tissue as compared with 6.77 ± 1.58 in normal hens). Expression of interleukin-16 was more than 2.5-fold higher and superoxide dismutase 2 was approximately 3-fold higher in PCOC hens than normal hens. The prospective study showed the development of OVCA in some hens with polycystic ovarian condition (PCO). PCOC development in hens was associated with chronic inflammation in the ovary. Laying hens may represent a potential model for the study of spontaneous PCOS and its long-term risk of PCOC development.

## 1. Introduction

Polycystic ovarian syndrome (PCOS) is a gynecological disorder affecting 5–10% women of reproductive age [1, 2]. PCOS is reported to be associated with endocrine and metabolic disorders leading to the manifestation of heterogeneous symptoms [3]. Although extensive studies have been performed, molecular etiology and long-term risks of PCOS remain controversial and largely unknown. Extensive studies further showed an association of PCOS with other serious conditions. Type 2 diabetes and cardiovascular diseases have been suggested as risk factors associated with PCOS [4], but whether additional long-term risks like the development of malignancies are associated with this syndrome is unknown. A few studies have suggested an association of PCOS with malignancies including ovarian cancer (OVCA) [5, 6] and endometrial cancer [7, 8]. Therefore, information on early etiologies as well as long-term risks of PCOS is urgently needed as it may not only save the life of patients but will also improve quality of life and save the burden of public health cost.

Hyperandrogenism, obesity with or without resistance to insulin, and ovarian dysfunction including oligo/anovulation are some of the symptoms associated with PCOS [9, 10]. Although endocrine imbalance is considered to be an etiology of PCOS, emerging information suggests that PCOS may also be an inflammatory event [11] and is associated with increased circulatory levels of several inflammatory markers [12]. However, obesity is also associated with low-grade inflammation [13]. Thus, it is possible that long-standing unresolved low-grade inflammation may be associated with the development of PCOS. However, it is unknown whether low-grade inflammation is a cause or an effect of PCOS.

Ovarian tissues including ovarian surface epithelium (OSE) and fimbrial surface epithelium (FSE) are exposed (at the site of ovulatory rupture and the site of receiving the ovulated ovum, respectively) to chronic inflammatory agents as a result of ovulatory injuries. Ovulation-related insults/exposures lead to the infiltration of immune cells, including macrophages and other members of the innate immune system, into the postovulatory follicular tissues. Macrophages are a source of IL-16 [14], a chemoattractant cytokine which may attract CD4 and CD8 T cells to the site of ovulatory rupture, as reported for injuries in other tissues [15]. Thus, infiltration and accumulation of immune cells at the site of ovulatory rupture may lead to the increased demand of oxygen resulting in the development of hypoxic condition. Hypoxia may subsequently lead to the development of oxidative stress and chronic low-grade inflammation [16, 17]. Furthermore, chronic low-grade inflammation and oxidative stress have also been suggested to be potential factors for the development of carcinogenesis [18]. Thus, persistent oxidative stress as well as chronic inflammation may be a risk factor for ovarian malignant development. It is possible that chronic low-grade inflammation and oxidative stress may be common conditions prevalent in both the PCOS [19] and OVCA. However, it is unknown whether low-grade inflammation of the ovary is associated with the development of PCOS and whether PCOS is a risk factor for ovarian carcinogenesis. Studies with humans on the prevalence of low-grade chronic inflammation in PCOS as an etiology and pathogenesis of OVCA development are difficult and time-consuming. Animal models have long been used in the study of human diseases that are difficult to study in humans.

Rodent models neither develop PCOS nor OVCA spontaneously [20–22]. Induced PCOS and OVCA in rodent models do not represent spontaneous PCOS and OVCA in women. However, laying hens develop OVCA spontaneously, and the histopathology of ovarian tumors as well as dissemination of OVCA is similar to women [21, 23]. Moreover, as in women, ovarian functions in chicken are also regulated by gonadotropins, including FSH (follicle-stimulating hormone), LH (luteinizing hormone), and ovarian steroids. Thus, the goals of this study were (1) to explore if laying hens develop polycystic ovarian condition spontaneously and if so, whether it is associated with chronic low-grade inflammation and (2) to determine if hens with polycystic ovarian condition develop OVCA. These goals were examined in two studies including an exploratory study and a prospective study.

## 2. Materials and Methods

All studies and procedures were performed as per the protocol approved by the Institutional Animal Care and Use Committee (IACUC) for animal use.

### 2.1. Animals (Chickens)

#### 2.1.1. Exploratory Study

Three- to four-year-old laying strains of white Leghorn hens (*Gallus domesticus*) were maintained with standard husbandry practices under an IACUC approved protocol with the provision of a 14 h light and 10 h dark period and free access to water and feed. Egg laying rates of hens are used as a relative indicator of their ovulation rates and are recorded on a daily basis. With normal husbandry practices, the egg laying rates of a healthy white Leghorn hen is approximately 250 eggs/year. When egg production rate drops to 50% or less than that of a healthy hen, it is considered as low rates of laying [24]. A total of 130 hens with normal and low or irregular egg laying rates and with or without abdominal distention (an indicator of potential ovarian cancer-related ascitic fluid) were selected randomly. Around 10–20% hens of this age group have been reported to develop OVCA [21, 22, 24].

### 2.2. Gross Examination and Tissue Collection

Blood was taken from all hens before euthanasia, and serum samples were separated. Following euthanasia, hens were examined for gross ovarian status. The presence or absence of cyst and/or solid mass was recorded and photographed. Tumors, when present, were staged according to the gross metastatic status as reported previously [21]. Precisely, in the early stage of ovarian cancer, the tumor is limited to the ovary and may or may not be accompanied with ascites. However, in the late stage of OVCA, the tumor had metastasized to other organs beyond the peritoneal cavity and was accompanied with medium to profuse ascites. Tissues, including normal ovaries as well as ovaries containing cysts and tumors at the early or late stage were divided into several blocks, processed for paraffin, or frozen sectioned for routine histology, immunohistochemistry, Western blotting, and gene expression studies.

### 2.3. Histologic Evaluation

Paraffin sections were examined by light microscopy using routine stains (hematoxylin-eosin) as reported previously [21] and evaluated for normal, cystic, and/or malignant features indicative of different types of tumor.

### 2.4. Immunohistochemical Examination

Localization of macrophages and IL-16- (a proinflammatory cytokine-) expressing cells as well as the intensity of SOD2 (superoxide dismutase 2, an enzyme with antioxidant property) expression in healthy ovaries and polycystic ovaries with cancer (PCOC) at the early stage (PCOC-ES) and late stage (PCOC-LS) was performed using antimacrophage (Abcam, Cambridge, MA), IL-16 (Kingfisher Biotech Inc., Saint Paul, MN), or SOD2 (rabbit anti-SOD2/MnSOD, Abcam, Cambridge, MA) primary antibodies, respectively, using a standard ABC staining method (VECTASTAIN Elite ABC HRP Kit, Peroxidase, Universal, R.T.U., Burlingame, CA) as reported previously [25]. In addition, representative sections of PCOC were stained for Ki67 expression (anti-Ki67 antibodies, Abcam, Cambridge, MA), a marker of cell proliferation, to determine the proliferative potential of cells of ovarian cysts. For all immunohistochemical markers, first antibodies were omitted in control staining and immunoreactions were not observed on the control section.

The frequencies of macrophages and IL-16-expressing cells or the intensities of SOD2 expression in 5 *μ*m thick sections were determined using a light microscope attached to a digital imaging software (MicroSuite version 5; Olympus Corporation, Tokyo, Japan). Three to five regions containing a high population of immunopositive cells or strong immunostaining in a section per ovary were selected, and the population of macrophages and IL-16-expressing cells were counted, or the intensities of SOD2 staining were determined. The frequencies of macrophages and IL-16-expressing cells or intensities of SOD2 staining in a section were determined at an ×40 objective and ×10 ocular magnification as reported previously [25]. These estimates of immunopositive cells (macrophages and IL-16-expressing cells) or intensities of SOD2 staining were expressed in a 20 mm^2^ area of the tissues in normal ovaries or in polycystic ovaries with cancer at the early or late stage.

### 2.5. One-Dimensional (1D) Western Blot (WB)

Immunohistochemical expression of SOD2 proteins by normal ovaries and polycystic ovaries with cancers at early and late stages was confirmed by 1D-WB using the same antibodies mentioned above (1 : 1000 dilution). Immunoreactions on the membrane were visualized as a chemiluminescence product (Super Dura West substrate; Pierce/Thermo Fisher, Rockford, IL), and images were captured using a ChemiDoc XRS (Bio-Rad, Hercules, CA).

### 2.6. Semiquantitative and Quantitative Polymerase Chain Reaction (RT-PCR/qRT-PCR)

Changes in the expression of IL-16 mRNA with regard to the development of OVCA in hens with polycystic ovaries were assessed by semiquantitative reverse transcription-polymerase chain reaction (PCR) as reported previously [25]. For reverse transcription-PCR analyses, representative samples from normal ovaries and polycystic ovaries with cancer at the early or late stage were selected based on their reactivity in immunohistochemistry. Hen-specific IL-16 primers were designed by OligoPerfect Designer software (Thermo Fisher Scientific, Waltham, MA) using the IL-16 sequence from the NCBI (GenBank NM_204352.3). The forward primer was 5-TCTCTGCTTTCCCCTGAA-GA, and the reverse primer was 5-GTCCATTGGGAAACACCT-TG, located between exons 4 and 6. *β*-Actin was used as the endogenous control with a forward primer of TGCGTGACATCAAGGAGAAG and a reverse primer of ATGCCAGGGTACATTGTGGT. The expected size of IL-16 amplicon was 199 base pairs, and it was 300 base pairs for *β*-actin. PCR amplicons were visualized in a 3% agarose gel (Pierce/Thermo Fisher) in Tris-acetate-EDTA buffer and stained with ethidium bromide. The image was captured using a ChemiDoc XRS system (Bio-Rad, Hercules, CA). Quantitative expression of IL-16 gene was examined using real-time PCR (TaqMan Gene Expression Assays, Applied Biosystems™, Thermo Fisher Scientific, Waltham, MA).

### 2.7. Prospective Study

Thirty-six laying hens of 3–4 years old (*n* = 36) reared under similar condition as mentioned earlier, without cysts or no solid mass in the ovary, were selected by transvaginal ultrasound (TVUS) scanning. Selected hens were reared under similar condition as mentioned above and monitored prospectively for 40 weeks with TVUS scanning at 10-week intervals as reported earlier [21]. Hens were euthanized upon diagnosis with the presence of solid mass in the ovary during prospective monitoring or at the end of the study period (40 weeks). Gross morphology was recorded as mentioned above.

### 2.8. Ultrasonography

Ovaries in hens were scanned using a commercially available sonography system attached to an endovaginal transducer (MicroMaxx® Ultrasound System, SonoSite Inc., Bothell, WA). Sonographic imaging was performed as reported previously [24, 26]. Briefly, transmission gel was applied on the surface of the endovaginal transducer and covered with a probe cover, followed by reapplying transmission gel on the probe cover. Two-dimensional gray-scale, color, and pulsed Doppler TVUS scanning was performed. Mechanical settings were optimized using fully functional ovaries in young laying hens and used as a control to tumor ovaries. The ovary and its neighboring areas were imaged by sweeping the transducer following the localization of a follicle. Evaluation of morphological features including ovarian cysts and solid masses, if present, was performed with gray-scale scanning, and attention was given to the number of cysts as well as developing hierarchical follicles, solid areas, and echogenicity. Once the morphological evaluations were completed, color Doppler was activated to detect vascular color signals and pulsed Doppler wave signals were recorded and used for the analysis of resistive indices. All TVUS scans (screenshots) were archived digitally as still format and real-time clips on one-sided recordable digital video disks (DVD+RW format; Maxell Corporation of America, Fair Lawn, NJ) readable on a personal computer as reported earlier [26].

### 2.9. Statistical Analysis

The differences in the frequencies of immunopositive cells or intensities of immunostaining among normal ovaries as well as polycystic ovaries with cancer at the early or late stage were analyzed using GraphPad Prism software (GraphPad Software Inc., San Diego, CA) and assessed by ANOVA and *F*-tests. Then, pairwise comparisons between the groups (normal ovaries or ovaries with cysts and cancer at the early or late stage) were performed by two-sample *t*-tests. Data are presented as mean ± SEM; all reported *P* values are 2-sided, and *P* < 0.05 was considered significant.

## 3. Results

### 3.1. Gross Examinations

**Figure 1:**
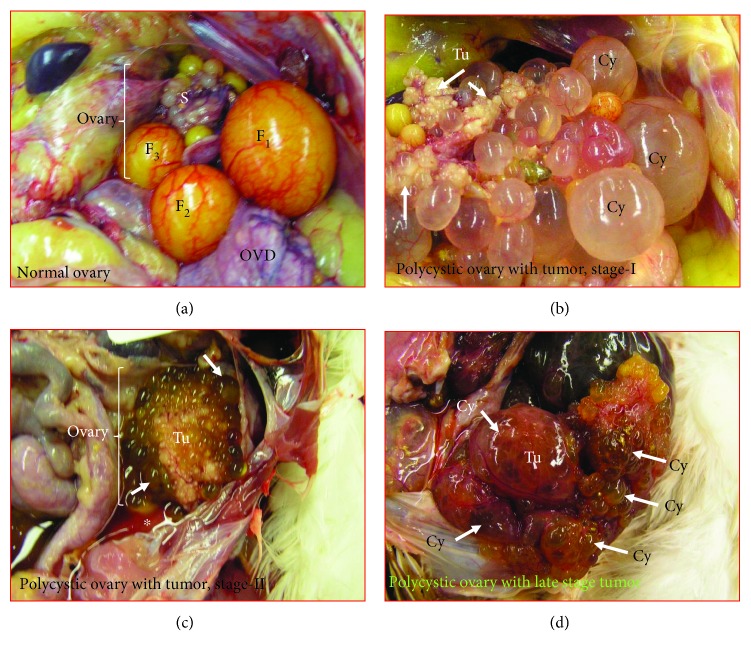
Gross presentations of normal ovaries and polycystic ovaries with cancer in hens. (a) An ovary in a healthy hen showing preovulatory follicles (F1–F3) and ovarian stalk (S) containing smaller growing follicles. (b) Polycystic ovary with cancer at stage I. The ovary contains numerous cysts (Cy) of different sizes, and solid tumor masses (Tu) are seen to be limited to few parts of the ovary (arrows). (c) Another case of polycystic ovary with cancer at stage II. The ovary contains numerous cysts (Cy) of different sizes (arrow), and a large solid tumor mass (Tu) is seen in the ovary accompanied with ascites (∗). Tumor metastasized to oviduct. (d) Polycystic ovary with cancer at the late stage. The tumor shows many cysts containing solid masses. The tumor metastasized to other distant organs. F1 = largest preovulatory follicle to be ovulated soon; F2 = second largest follicle; F3 = third largest follicle; OVD = oviduct.

In laying hens, the left ovary develops to a mature functional ovary whereas the right ovary becomes rudimentary. Chickens normally lay an egg each day for 5–6 days; after which, the hen pauses a day. Then, she resumes egg laying for 5 to 6 days (termed as the clutch size or laying sequence). Upon euthanasia, hens with normal and functional ovaries had 3–5 large preovulatory hierarchical follicles, several small yolky follicles, and many white follicles protruding from the ovarian surface with no cysts or solid tissue masses (Figure 1(a)). These ovaries were categorized as “*normal ovaries*.” Of 130 hens in the exploratory study, ovaries in 8 hens had fluid-filled ovarian cysts (number of cysts, *n* = >25) of varying sizes (small, medium, and large) protruding from the ovarian surface as well as solid ovarian mass either limited to a part of the ovaries (*n* = 4, early stage OVCA) or metastasized to distal organs (*n* = 4, late stage OVCA) (Figures 1(b) and 1(d)). One or two preovulatory follicles were occasionally detected in these ovaries. These ovaries were classified as *polycystic ovaries with cancer* (PCOC) at the early stage (PCOC-ES) and late stage (PCOC-LS). Tumors in these hens were accompanied with or without ascetic fluid. In addition, 19 hens without any ovarian cyst had a solid tissue mass either limited to a part of the ovary (early stage OVCA, *n* = 7) or metastasized to distal organs accompanied with profuse ascites (late stage OVCA, *n* = 12).

### 3.2. Microscopic Observations

**Figure 2:**
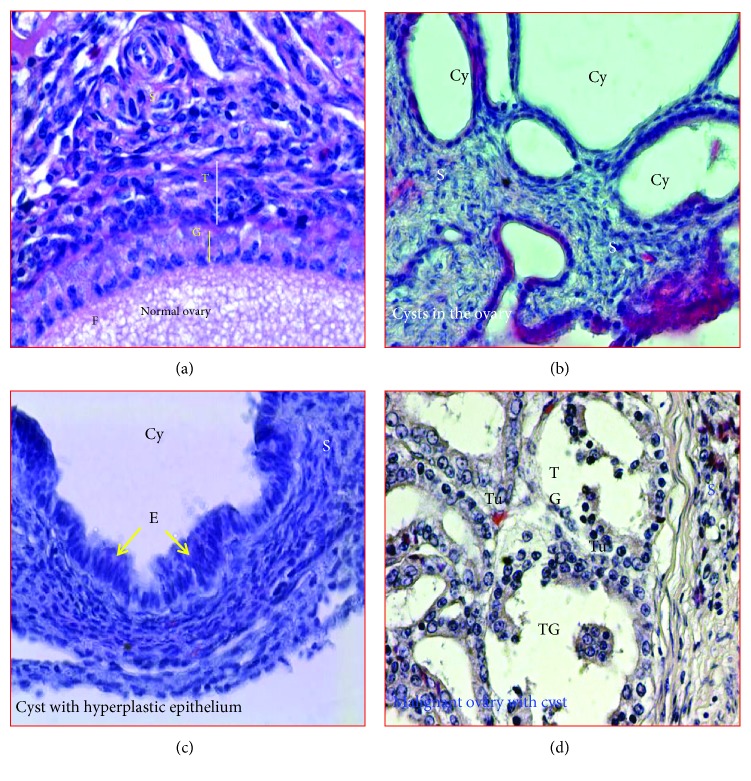
Microscopic presentations of normal ovary and polycystic ovaries with cancer at early and late stages stained with hematoxylin and eosin. (a) Section of normal ovary in a healthy hen showing embedded stromal follicle in the stroma. (b) Section of a polycystic ovary showing many cysts of different sizes and shapes in the stroma. (c) A cyst in the stroma of the ovary in the hen with polycystic ovary. Epithelial cells of the cyst show hyperplasia which is considered as precursor lesion for malignant transformation. (d) Polycystic ovary with cancer shown in Figure 1(b). The tumor is an endometrioid carcinoma with confluent back-to-back tumor glands. Cy = cyst; E = epithelial cells; F = follicle; G and T = granulosa and theca layers of the stromal follicle; S = stroma; TG = tumor gland. Magnification = 40x.

From normal hens with functional ovaries, 20 hens were selected randomly for microscopic evaluation. Stromal embedded follicles including primordial and primary follicles containing the developing oocytes surrounded by granulosa and undifferentiated theca layers and few atretic follicles were observed in the ovaries of normal hens (Figure 2(a)). In hens with PCOC at early and late stages, multiple ovarian cysts with or without oocytes were observed to be embedded in the stroma (Figure 2(b)). Compared with normal ovaries, many embedded atretic follicles were also seen in these ovaries. In a few cases, stromal cysts were observed to contain hyperplastic cells with mitotic figures suggestive of potential malignant transformation (Figure 2(c)). Malignant cells in PCOC hens were large in size with pleomorphic nuclei containing multiple mitotic figures (Figure 2(d)).

### 3.3. Expression of Markers of Cell Proliferation

**Figure 3:**
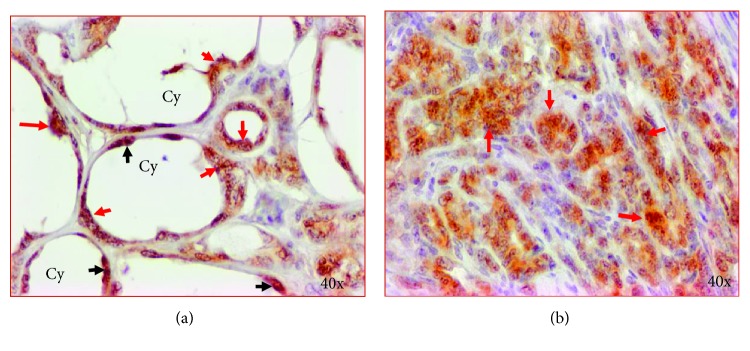
Expression of Ki67, a marker of cell proliferation, by the epithelial cells in ovarian cysts and malignant cells in hen ovaries. (a) Intense staining for Ki67 was shown by cells of ovarian cysts, some of which appeared as a cluster of cells (red arrows) while a few normal appearing cells also stained (black arrows). (b) A section from a PCOC showing intense staining for Ki67 shown by the malignant cells (red arrows) similarly shown by the cyst. Cy = cyst. 40x = magnification.

Uncontrolled cell proliferation is a hallmark of cancer and Ki67, a marker of cell proliferation which is commonly used to determine cell proliferation during malignant transformation. To examine if cysts in hens with polycystic ovarian conditions and ovarian cancer express Ki67, sections were stained with Ki67. Intense expression of Ki67 by malignant cells of PCOC was observed (Figure 3). Similar patterns of intense Ki67 expression were also detected in the epithelial cells of cysts. Some of these cells in the cysts were arranged in small cluster-like morphology (red arrows), whereas some had normal appearing monolayer of epithelial cells (black arrows) (Figure 3).

### 3.4. Localization of Macrophages

**Figure 4:**
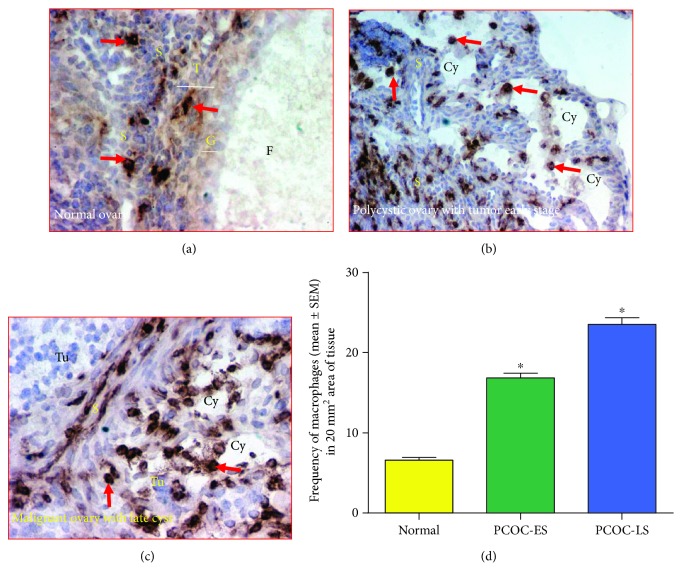
Localization of macrophages in normal ovaries and polycystic ovaries with cancer. (a) Section of a normal ovary. Very few macrophages are seen in the stroma and theca layer of the stromal embedded follicle. (b) Section of a polycystic ovary with cancer at the early stage (PCOC-ES). Many macrophages are seen in the stroma containing the tumor. Macrophages are also localized in and around the wall of cysts. (c) Section of a polycystic ovary with ovarian carcinoma at the late stage (PCOC-LS). Many macrophages are localized in the stroma containing cysts. (d) Changes in the frequency of macrophages in polycystic ovaries in association with ovarian malignant development and progression. Compared with normal ovaries, the frequency of macrophages increased remarkably in polycystic ovaries with cancer at the early stage (*P* < 0.0001). The frequency of macrophages increased further as the tumor progressed to late stages (*P* < 0.0001). Arrows indicate examples of immunopositive cells. Cy = cyst; F = follicle; G and T = granulosa and theca layers of the stromal follicle; S = stroma; Tu = tumor. Magnification = 40x.

Cells stained with antimacrophage antibodies were irregular in shape and localized in the stroma and the theca layer of follicles in normal ovaries (Figure 4(a)). In hens with PCOC at early and late stages, macrophages were localized in the stroma as well as in the vicinity of ovarian cysts (Figures 4(b) and 4(c)). Compared with normal ovaries, many macrophages were localized in the stroma surrounding the tumor and the cysts (Figures 4(b) and 4(c)). In addition, compared to the theca layers in normal stromal follicles, many macrophages were also observed in ovarian cysts.

Compared with the frequency of macrophages in hens with normal ovaries (6.77 ± 1.58 macrophages in a 20 mm^2^ area of tissue), the frequency of macrophages was significantly greater (*P* < 0.0001) in the stroma of PCOC at the early stage (17.33 ± 2.26 macrophages in a 20 mm^2^ area of tissue) and increased further in PCOC at the late stage (24.24 ± 2.50 macrophages in a 20 mm^2^ area of tissue) (Figure 4(d)).

### 3.5. Detection of IL-16-Expressing Cells

**Figure 5:**
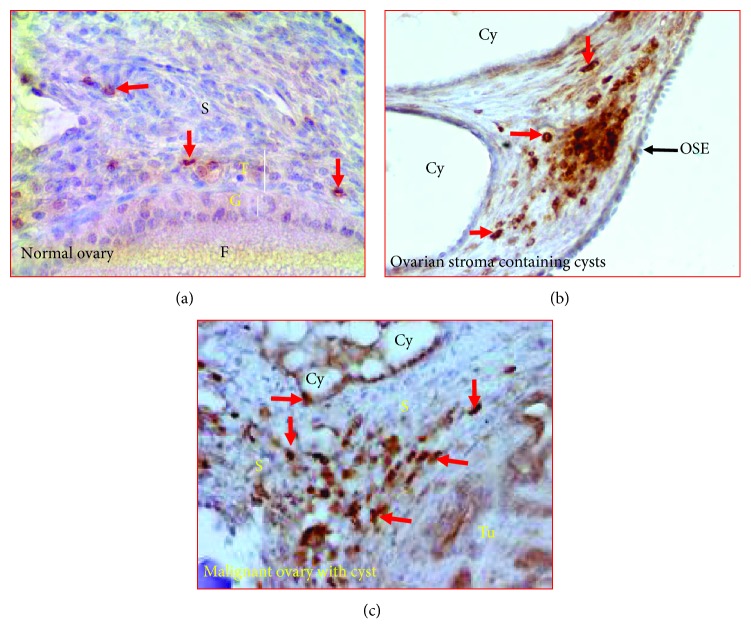
Immunohistochemical detection of IL-16-expressing cells in polycystic ovaries with cancer in hens. (a) Section of a normal ovary. Very few IL-16-expressing cells are seen in the stroma and theca layer of the stromal follicle. (b) Section of ovarian stroma containing cysts shows influx of IL-16-expressing cells in the vicinity of cysts in the stroma. (c) Section of a polycystic ovary with ovarian carcinoma. Many IL-16-expressing cells are seen in the stroma containing cysts as well as tumor glands. Arrows indicate examples of immunopositive cells. Cy = cyst; F = follicle; G and T = granulosa and theca layers of the stromal follicle; OSE = ovarian surface epithelium; S = stroma; Tu = tumor. Magnification = 40x.

Few IL-16-expressing cells were detected in the stroma of normal ovaries (Figure 5(a)). Influx of IL-16-expressing cells was detected in the vicinity of ovarian cysts in the stroma (Figure 5(b)). Moreover, many IL-16-expressing cells were localized in the stromal tissue surrounding the cysts and tumor in PCOC hens (Figure 5(c)). Occasional staining for IL-16 by malignant cells as well as cells of ovarian cysts was also seen in hens with PCOC (Figure 5(c)).

**Figure 6:**
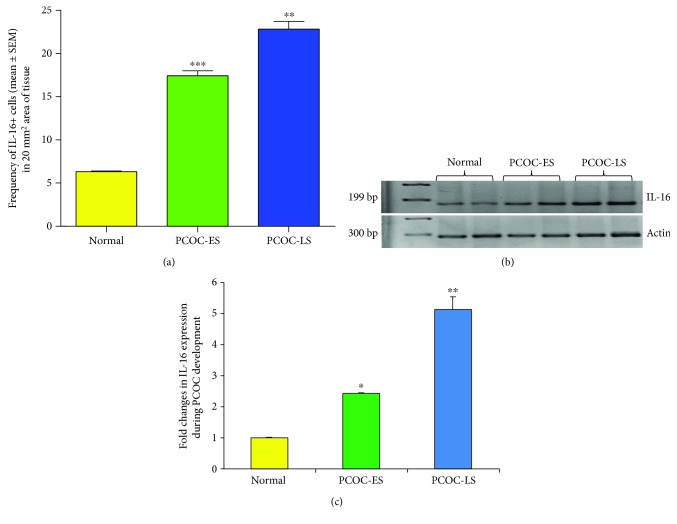
Changes in the frequency of IL-16-expressing cells in polycystic ovaries during the development and progression of cancer. (a) Compared with normal ovaries, the frequency of IL-16-expressing cells was significantly high in polycystic ovaries with cancer at the early stage (PCOC-ES) (*P* < 0.0001) and increased further in PCOC at the late stage (PCOC-LS) (*P* < 0.008). (b, c) Gene expression studies for IL-16 (semiquantitative (b) and quantitative PCR (c)). Compared with normal, IL-16 gene expression was stronger (b) and significantly higher in PCOC-ES and increased further in PCOC-LS (c) confirming the observations of immunohistochemical studies.

Compared with the frequency of IL-16-expressing cells in normal hens (6.32 ± 1.72 in a 20 mm^2^ area of tissue), the frequency of stromal IL-16-expressing cells was significantly higher (*P* < 0.0001) in hens with PCOC at the early (17.43 ± 3.91 in a 20 mm^2^ area of tissue) and late stages (22.83 ± 4.37 in a 20 mm^2^ area of tissue) (Figure 6(a)).

Tissue expression of IL-16 detected by immunohistochemistry was confirmed with gene expression studies including semiquantitative and real-time PCR. As observed in immunohistochemical studies, strong amplification for IL-16 mRNA expression was detected in PCOC at early and late stages (Figure 6(b)). These observations were further confirmed by quantitative PCR which showed significant increase in IL-16 gene expression in PCOC at early and late stages (Figure 6(c)).

### 3.6. Intensity of SOD2 Expression

**Figure 7:**
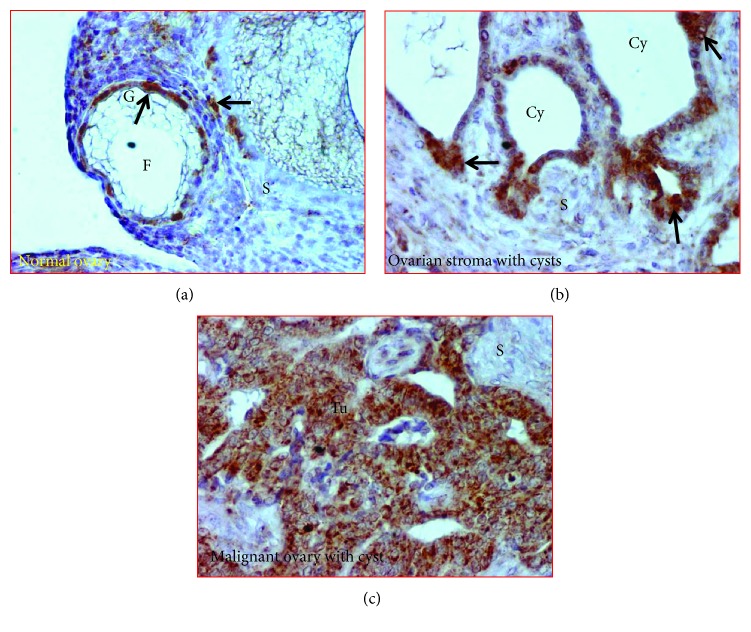
Expression of superoxide dismutase 2 (SOD2) in normal ovaries or polycystic ovaries with cancer. (a) Section of a normal ovary. Few granulosa (G) cells of the stromal follicle and few stromal cells stained for SOD2. (b) Section of an ovarian stroma with cysts showing a strong expression of SOD2 by the epithelial cells of the cysts. (c) Section of a polycystic ovary with ovarian cancer. Intense expression of SOD2 was observed in the polycystic ovary with cancer. Arrows indicate examples of immunopositive cells. Cy = cyst; F = follicle; G = granulosa layer of the stromal follicle; S = stroma; Tu = tumor. Magnification = 40x.

Expression of SOD2 was located in the cytoplasm of epithelial as well as stromal cells in normal ovaries, in ovarian cysts, and malignant cells in PCOC at early and late stages (Figures 7(a) and 7(c)). SOD2 staining was observed in the stroma and granulosa cells of stromal follicles in normal ovaries (Figure 7(a)). However, the staining for SOD2 expression was stronger in the cells of ovarian cysts (Figure 7(b)) and malignant cells in PCOC (Figure 7(c)).

**Figure 8:**
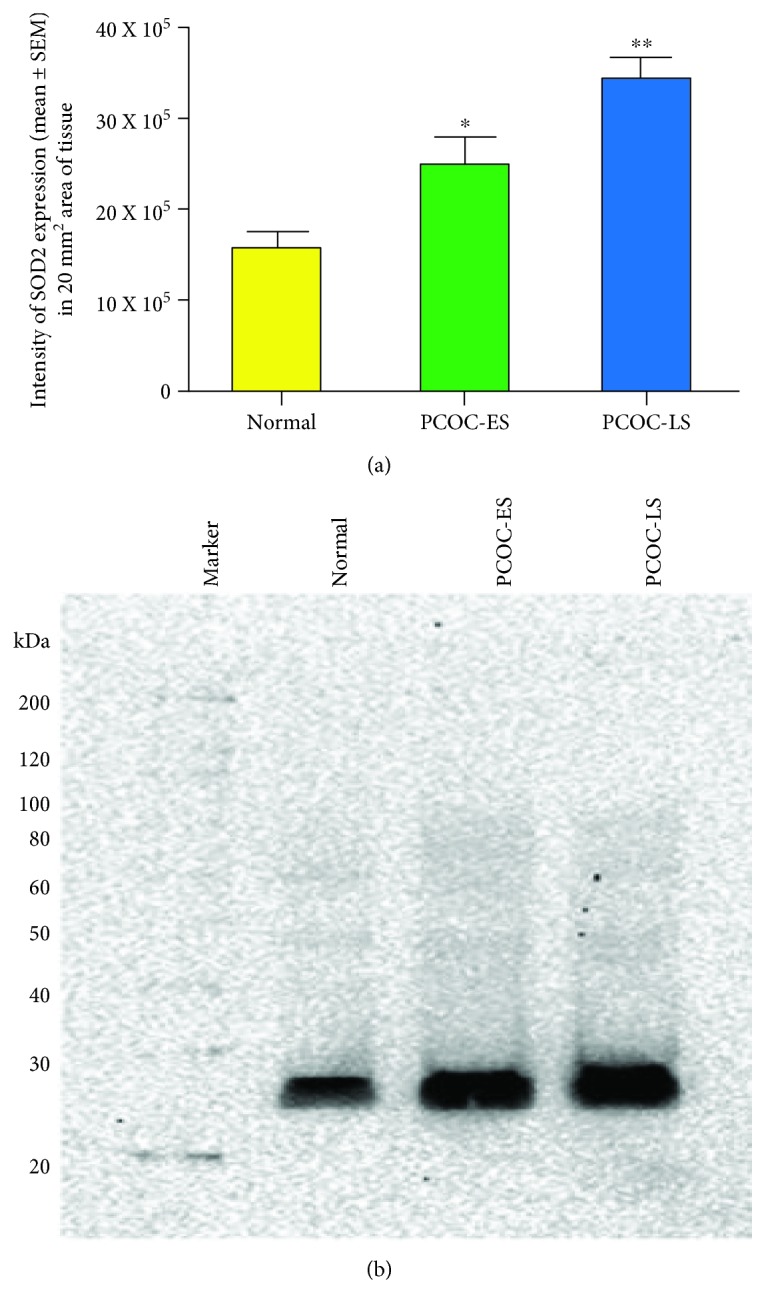
Changes in the intensity of SOD2 expression in polycystic ovaries during the development and progression of cancer. (a) Compared with normal ovaries, the intensity of SOD2 expression was significantly higher in polycystic ovaries with cancer at the early stage (PCOC-ES) (*P* < 0.04) and increased further in PCOC at the late stage (PCOC-LS) (*P* < 0.006). (b) Immunoblotting showed a band for SOD2 at approximately 27 kDa and confirmed the increasing patterns of SOD2 expression in relation to tumor development and progression (to PCOC-ES and PCOC-LS as observed in immunohistochemistry).

Overall, the intensity of SOD2 expression increased significantly (*P* < 0.0001) with the progression of the disease from normal to PCOC-ES to PCOC-LS. Compared with the intensities of SOD2 expression in normal hens (15.7 × 10^5^ ± 6.3 × 10^5^ in a 20 mm^2^ area of tissue), the intensity of SOD2 staining (24.9 × 10^5^ ± 14.3 × 10^5^ in a 20 mm^2^ area of tissue) was significantly higher in hens with PCOC at the early stage (*P* < 0.04). The intensity of SOD2 staining increased further (34.7 × 10^5^ ± 10.5 × 10^5^ in a 20 mm^2^ area of tissue) in hens with PCOC at the late stage (*P* < 0.0006) (Figure 8(a)).

Immunoblotting was performed to confirm the immunohistochemical expression of SOD2 by ovarian tissues. A band size of approximately 27 kDa was detected in ovarian homogenates (Figure 8(b)). Compared with normal ovaries, the expression of SOD2 was stronger in the homogenates of polycystic ovaries with cancer at the early stage (PCOC-ES) and the late stage (PCOC-LS) (Figure 8(b)).

### 3.7. Evaluation of Prospective Sonographic Monitoring

**Figure 9:**
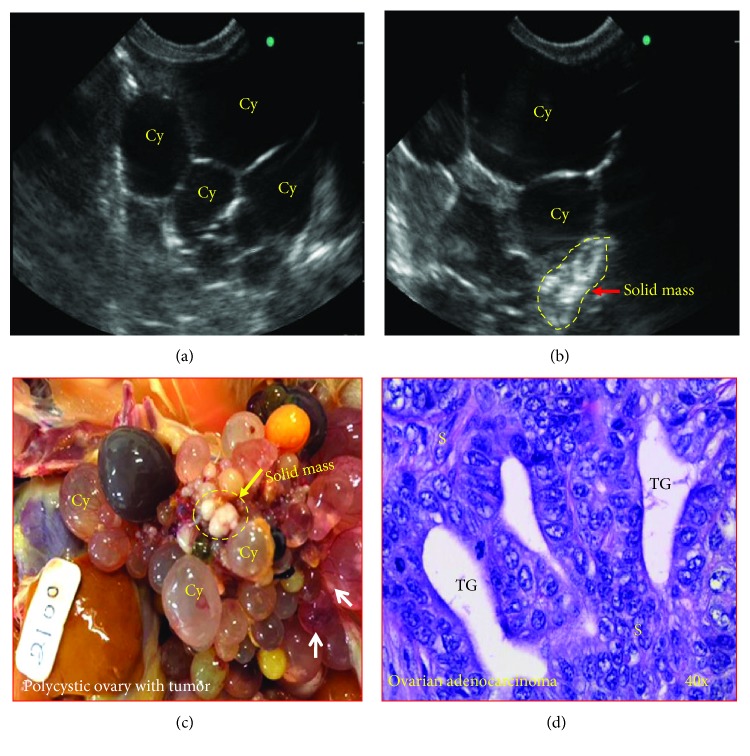
Prospective monitoring of hens with polycystic ovaries to detect malignant development by transvaginal ultrasound imaging. (a) Gray-scale sonogram of an ovary in a hen. Multiple cysts of various sizes and shapes are seen during ultrasound monitoring. (b) Sonogram of the same hen after 20 weeks. Many cysts are seen in the ovary. During monitoring period, small solid masses are seen to be developed in the ovary. (c) Gross presentation of the ovary sonogram which is presented in (b). Gross examination confirmed the development of tumor in the ovary (yellow circle). Many cysts of different sizes are also present. White arrows show the formation of solid mass inside the cysts. (d) Microscopic examination confirmed that the tumor was an endometrioid ovarian carcinoma. Cy = cyst; S = stroma; TG = tumor gland.

During prospective monitoring, gray-scale sonographic morphology of large preovulatory follicles showed a dark circular or oval oocyte containing a concentric ring at the center of each follicle. As compared with functional preovulatory follicles, ovarian cysts were of different sizes, fluid-filled, and irregularly shaped with a relatively thin and sharp cyst wall without any concentric ring (Figures 9(a) and 9(b)). During prospective monitoring, 2 hens with polycystic ovaries showed small solid tissue masses limited to a part of the ovaries (Figures 9(a) and 9(b)). These hens were provisionally diagnosed to have PCOC. On the other hand, 3 hens were observed to have a solid mass in the ovaries without any cysts, and these hens were provisionally diagnosed to have OVCA. Overall, gross examinations following euthanasia of hens at the end of the monitoring period confirmed the ultrasound prediction that 2 of 36 hens had PCOC (Figure 9(c)) while 3 hens had ovarian tumors with no cysts in the ovary. Following gross evaluation, tumors were also confirmed upon with routine histology as mentioned earlier (Figure 9(d)).

## 4. Discussion

This is the first study reporting the spontaneous incidence of polycystic ovarian condition in laying hens, a preclinical model of spontaneous ovarian cancer. This exploratory part of this study found that approximately 6% of hens with ovarian tumors had polycystic ovarian condition, suggesting that polycystic ovarian condition may be a potential risk factor for the development of spontaneous ovarian cancer. This assumption is based on the results of the prospective monitoring of hens in which a subset of them with polycystic ovaries developed spontaneous ovarian cancer. The results of this study also suggest the prevalence of low-grade chronic inflammation during the development and progression of spontaneous ovarian cancer in hens with polycystic ovarian condition. This study further suggests that persistent oxidative stress may be associated with ovarian low-grade inflammation, and persistent oxidative stress in polycystic ovaries may be a reason for the malignant transformation of these ovaries.

Ovaries in women with PCOS contain multiple cysts [27]. In this study, similar to women, hens with polycystic ovaries and cancer at the early stage had more than 25 fluid-filled cysts of varying sizes in their ovaries. Furthermore, polycystic ovarian condition in hens was accompanied with fewer (only one or two) preovulatory functional (ovulable) follicles compared with their normal counterpart hens. Thus, egg laying rates (an indicator of ovulation rates) in these hens were also remarkably lower, indicating that polycystic ovarian condition in hens is associated with the reduced rates of ovulation and egg laying. Therefore, the presence of multiple cysts together with reduced egg laying rates may suggest defects in ovarian functions including follicular growth and/or ovulation in these hens. Endocrine imbalance may be one of such defects involved in the reduction of ovarian function, as suggested in humans [28]. The basic physiological processes of ovarian functions including follicular growth, maturation, and ovulation are controlled by endocrine regulations in hens and in humans [29]. Furthermore, emerging information indicates the potential association of additional nonendocrine conditions including chronic inflammation of the ovary with the incidence of PCOS in women. In addition, chronic inflammation and unresolved oxidative stress are predisposing factors for malignant transformation [18]. This study showed that, compared with normal ovaries, the frequencies of macrophages and IL-16- (a proinflammatory and chemotactic cytokine-) expressing cells were higher in polycystic ovaries with cancer at the early stage and late stage. In addition, influx of IL-16 was detected in close proximity of ovarian cysts in the stroma. Moreover, immunoreaction for IL-16 expression was also detected in the epithelial cells (inner lining wall) of the cysts in the stroma. IL-16 is a proinflammatory cytokine secreted by macrophages, CD8 T cells, and epithelial cells [14, 30, 31]. IL-16, a chemoattractant, leads to the homing of other immune cells at the site of injury and inflammation, resulting in an oxidative burst which subsequently causes oxidative stress [32, 33]. These results suggest that a condition of low-grade inflammation is associated with the development of polycystic ovarian condition. Chronic inflammation is the consequence of chronic activation of immune cells and an increase in secretion of proinflammatory cytokines and chemokines including IL-18, monocyte chemoattractant protein-1 (MCP-1), and macrophage inflammatory protein-1*α* (MIP-1*α*) [34–36]. Prevalence of a low-grade inflammatory condition was reported in women with polycystic ovarian syndrome in which ovaries were infiltrated by macrophages [19, 37, 38]. This study also showed an increase in the population of macrophage and its secretory proinflammatory product, IL-16-expressing cells, in association with PCOC ovaries. However, it is not known whether a low-grade inflammatory condition is required for the development of polycystic ovarian condition.

The assumption that persistent oxidative stress and chronic low-grade inflammation are predisposing factors for the development of polycystic ovarian condition and the subsequent development of ovarian cancer is based on the observation of an increased expression of SOD2 by the hen ovaries. The current study observed the expression of antioxidant enzyme, SOD2, by the cells of ovarian cysts and malignant cells in PCOC at early and late stages. SOD2 plays important roles in neutralizing reactive free oxidative radicals. An influx of immune cells to the site of chronic injury (e.g., ovulatory injuries in the ovary) and the subsequent development of oxidative stress may lead to chronic inflammation [11]. During oxidative stress, reactive oxygen species (ROS) including superoxides are produced as byproducts of the mitochondrial electron transport chain [39]. Superoxides are highly toxic causing premature cell death, and cells increase the expression of SOD2 to withstand oxidative stress [40, 41]. SOD2 scavenges superoxides and converts them to hydrogen peroxide and diatomic oxygen and thus, protects cells from death [42]. Thus, enhanced SOD expression is a surrogate marker of oxidative stress as well as inflammation.

Increased expression of SOD2 in ovarian cysts and tumors in PCOC hens observed in this study may be a response to the oxidative stress due to an increased infiltration of immune cells (macrophages and IL-16-expressing cells) in polycystic ovaries and the subsequent development of PCOC. Macrophages have been shown to be infiltrated into the ovaries with PCOS [11], and SOD2 facilitates survival of tumor cells from oxidative stress-related death in the tumor microenvironment, including ovarian clear cell carcinoma [43] and neuroendocrine tumors [44]. Furthermore, macrophages have been implicated to be associated with malignant progression. IL-16 has been reported to translocate from the cytoplasm to the nucleus where it stimulates the expression of MDM2, an inhibitor of tumor suppressor p53. Inhibition of p53 may lead to the uncontrolled growth of abnormal cells leading to malignancy [45]. IL-16 has also been shown to be associated with malignancies including leukemia and ovarian cancer development and progression [46]. Endocrine-immune interaction has been reported to be involved in the regulation of normal and abnormal ovarian functions. Thus, in the context of chronic and long-standing unresolved inflammation, members of the immune system may modulate endocrine physiology of the ovary leading to the development of polycystic ovarian condition, which may be involved in the subsequent development of ovarian cancer in hens. This assumption is also supported by the similarities in the expression of the marker of cell proliferation and malignant transformation (Ki67) by the cells of cysts.

This study has several translational aspects. The lack of an easily accessible spontaneous model is a significant barrier to the generation of information on the etiopathogenesis of polycystic ovarian condition and PCOC. The lack of a preclinical model of spontaneous ovarian cancer also affects the studies which are aimed at generating information required for the development of interventional strategies for polycystic ovarian condition and ovarian cancer. Rodent models of induced polycystic ovarian condition do not represent that of spontaneous PCOS. PCOS in humans is a condition with a multifactorial etiology and is heterogeneous in nature. Moreover, rodent models do not develop long-term risks of ovarian pathology including spontaneous ovarian cancer. Thus, observations from rodent models, including hormonal induction and genetic mutations, are difficult to translate to the clinics for the study of the risk of polycystic ovarian condition with the subsequent development of ovarian cancer. In contrast, as observed in our exploratory studies followed by the prospective studies, the laying hen develops polycystic ovarian condition spontaneously with features similar to those observed in humans (including multiple fluid-filled cysts, decreased rates of ovulation, and persistent low-grade inflammation). Furthermore, OVCA in hens also expresses several markers similarly expressed in human OVCA. In addition, similarities also exist in endocrine control of ovarian functions in hens and humans. Moreover, laying hens are widely available, easily accessible, cost-effective compared to rodents, and easy to manage. Besides, the availability of noninvasive transvaginal ultrasound scanning for prospective monitoring also facilitates the feasibility of laying hens as a model for the generation of information on the spontaneous development of polycystic ovarian condition and PCOC, which is difficult to study in humans.

This study has also some limitations including a smaller number of samples as well as the length of prospective monitoring of hens for the development of polycystic ovarian condition and the subsequent progression to ovarian cancer. First, although PCOS in women is a complex disease with heterogeneous etiologies, some established and accepted guidelines are available for PCOS diagnosis. As this is the first study on PCO in hens, unfortunately no such criteria for the diagnosis of PCO in hens are available. However, this study showed several features associated with PCO including number of cysts, increased frequency of macrophages, oxidative stress, and inflammation in hens similarly reported in humans with PCOS [11, 27, 38]. Second, additional features of PCOS in human including development of insulin resistance and hyperandrogenism have not been studied in hens. However, these features in human PCOS have been shown to be the consequences of oxidative stress [11], and this study also showed increased oxidative stress in hens with PCOC. Third, one of the limitations of this study is that it examined normal hens and PCO hens with early and late stage OVCA, but it did not study hens with only PCO without an ovarian tumor. However, the prospective part of this study mitigated this limitation as it monitored hens with only PCO diagnosed by ultrasound scanning to determine the development of OVCA in PCO hens. Fourth, this study used SOD2 as a marker of oxidative stress, which may not be a strong and reliable marker of oxidative stress. However, SOD2 has been used as a marker of oxidative stress and has been shown to prevent oxidative stress [41]. Besides, SOD2 has also been reported to facilitate tumor metastasis [27, 43]. Moreover, SOD2 was also chosen as it inhibits the expression of inflammatory cytokines [47]. In lieu of all these limitations, the observations of this study will lead to the establishment of the laying hens as a model of spontaneous polycystic ovarian condition to study its long-term risks including OVCA. Furthermore, this model will also be useful for the study on the development of preventive measures against the incidence of polycystic ovarian condition and its subsequent risk of ovarian cancer.

## 5. Conclusions

In conclusion, the results of this study suggest that laying hens develop polycystic ovarian condition spontaneously, which is associated with chronic inflammation and oxidative stress. As the inflammation and chronic stress are hallmarks of malignant development, PCO may be a potential risk factor for subsequent development of ovarian cancer. This assumption is based on our observation from prospective monitoring of PCO hens which developed OVCA later. Thus, the laying hen offers a potential model to generate information on the etiology of spontaneous incidence of polycystic ovarian condition and its long-term risk of ovarian cancer development. This model will also be useful to develop strategies to prevent the incidence of polycystic ovarian condition and the risk of OVCA development.

## Data Availability

The data used to support the findings of this study are available from the corresponding author upon request.
